# A Novel Apilic Antivenom to Treat Massive, Africanized Honeybee Attacks: A Preclinical Study from the Lethality to Some Biochemical and Pharmacological Activities Neutralization

**DOI:** 10.3390/toxins13010030

**Published:** 2021-01-05

**Authors:** Jhonatha Mota Teixeira-Cruz, Marcelo Abrahão Strauch, Marcos Monteiro-Machado, Matheus Silva Tavares-Henriques, João Alfredo de Moraes, Luís Eduardo Ribeiro da Cunha, Rui Seabra Ferreira, Jr., Benedito Barraviera, Luis Eduardo M. Quintas, Paulo A. Melo

**Affiliations:** 1 Graduate Program in Pharmacology and Medicinal Chemistry, Institute of Biomedical Sciences, Federal University of Rio de Janeiro, Rio de Janeiro 21941-902, Brazil; jhonatha_cox@hotmail.com (J.M.T.-C.); marcosmmachado@gmail.com (M.M.-M.); matheus-sth@hotmail.com (M.S.T.-H.); jmoraesbr@yahoo.com (J.A.d.M.); 2 Scientific Board, Vital Brazil Institute (IVB), Niterói, Rio de Janeiro 24230-410, Brazil; luis.eduardo@vitalbrazil.rj.gov.br; 3 Center for the Study of Venoms and Venomous Animals (CEVAP), São Paulo State University (UNESP), Botucatu, São Paulo 18610-307, Brazil; rui.seabra@unesp.br (R.S.F.J.); bbvieira@gmail.com (B.B.)

**Keywords:** Apilic antivenom, *Apis mellifera*, honeybee venom, melittin, preclinical study, envenomation

## Abstract

Massive, Africanized honeybee attacks have increased in Brazil over the years. Humans and animals present local and systemic effects after envenomation, and there is no specific treatment for this potentially lethal event. This study evaluated the ability of a new Apilic antivenom, which is composed of F(ab’)2 fraction of specific immunoglobulins in heterologous and hyperimmune equine serum, to neutralize *A. mellifera* venom and melittin, in vitro and in vivo, in mice. Animal experiments were performed in according with local ethics committee license (UFRJ protocol no. DFBCICB072-04/16). Venom dose-dependent lethality was diminished with 0.25–0.5 μL of intravenous Apilic antivenom/μg honeybee venom. In vivo injection of 0.1–1 μg/g bee venom induced myotoxicity, hemoconcentration, paw edema, and increase of vascular permeability which were antagonized by Apilic antivenom. Cytotoxicity, assessed in renal LLC-PK1 cells and challenged with 10 μg/mL honeybee venom or melittin, was neutralized by preincubation with Apilic antivenom, as well the hemolytic activity. Apilic antivenom inhibited phospholipase and hyaluronidase enzymatic activities. In flow cytometry experiments, Apilic antivenom neutralized reduction of cell viability due to necrosis by honeybee venom or melittin. These results showed that this antivenom is effective inhibitor of honeybee venom actions. Thus, this next generation of Apilic antivenom emerges as a new promising immunobiological product for the treatment of massive, Africanized honeybee attacks.

## 1. Introduction

The honeybees *Apis mellifera* has great worldwide economic and ecological importance, involving mainly plant pollination and honey and derivatives production [1,2]. The European honeybee (*A. mellifera*) was introduced to North and South America from Old World populations in the early 18th century [3]. 

In 1956, a new hybrid bee first appeared in Brazil after a laboratory incident caused by the cross between a European *A. mellifera mellifera* and *A. mellifera scutellata* from Africa [4,5]. This hybrid has spread gradually over the years to several American countries, including the United States [6,7]. 

According to Kadri et al. [4], there is a growing need to quickly and reliably detect these new hybrid Africanized colonies because they are not only extremely defensive, prone to massive attacks and highly invasive, but also within the last 60 years have become the most common genotype across much of the southern United States [4,5,6,7].

The increase of envenomation cases caused by the Africanized honeybees has been related to the emergence and expansion of these insects throughout the Americas [8,9,10,11]. When threatened, these insects become aggressive and can attack massively, causing serious and occasionally fatal cases [12,13,14]. On a temporal scale, over the years the number of cases reported in Brazil has increased exponentially, as demonstrated by epidemiological studies [15,16] and data from the Brazilian National Disease Notification System (SINAN) [17]. Between the years 2000 and 2017, the number of notifications raised by 11-fold (from 1440 to 17,078 cases, respectively) [17].

Africanized *A. mellifera* whole venom is composed of several substances, including the main toxin melittin (50%), phospholipase A_2_ (PLA_2_) (10–12%), hyaluronidase (1–3%), and apamine (1–3%), among other components [18]. Multiple stings inject several micrograms of bee venom and melittin. The plasma half-life for melittin is around 30 min and it is rapidly sequestered in the lungs, spleen, liver and erythrocytes [19].

Envenomation response varies from individual to individual and depends on several factors including the number of stings and patient’s allergic status. In some cases, the response is limited to the site of the sting where there is formation of painful papules, edema, erythema and local burning pain caused by increased vascular permeability, which begin to disappear within 24 h. About 50% of the cases evolve into larger edematous local reactions similar to cellulitis or the lesion may become vesicular or bullous. Some patients have systemic allergic reactions (almost 4% in the United States), including severe anaphylaxis [20,21].

The toxic reactions from multiple stings generate severe systemic disturbances, such as hemolysis, rhabdomyolysis, acute renal failure, myocardial infarction; hepatocellular necrosis, respiratory distress and multiorgan failure [22,23,24]. The inflammatory process may be due to the direct (mast cell degranulating peptide) or indirect (PLA_2_, hyaluronidase) action of substances present in the venom and the release of pro-inflammatory mediators from mast cell degranulation, tissue injury or immune responses [8,25].

Although antivenoms were developed decades ago to treat snake, spider, and scorpion envenomations, currently, there is no clinically approved antivenom against massive honeybee attacks. Thus, the official therapeutic guidelines are only to support and relieve the signs and symptoms [26].

To solve this problem, over the last two decades a task force from a translational research center of a Brazilian public university (Center for the Study of Venoms and Venomous Animals (CEVAP from São Paulo State University (UNESP), São Paulo, Brazil, in partnership with a traditional serum-producing Institute (Vital Brazil Institute) developed a new Apilic antivenom (ApAV) to treat massive, Africanized honeybee attacks. ApAV is a formulation containing polyvalent equine F(ab’)2 fractions raised against bee venom toxins. The clinical trial phase I/II finished in 2019 demonstrated that it is a safe product with promising efficacy [14]. ApAV was produced from the immunization of horses exclusively with two main toxins purified from the venom of Africanized honeybees, melittin and PLA_2_ (EC: 3.1.1.4), with the objective of minimizing their lethal activity in patients, being the most efficient due to its antivenom specificity.

The present study aimed to preclinically evaluate the neutralizing activity of ApAV by using different approaches which were chosen due to the specific in vivo (local vascular permeability, edema, hemoconcentration, myodegeneration, and death) and in vitro (myeloperoxydase, phospholipase A2, hyaluronidase, and cytotoxic activity) effects of the venom and melittin from the Africanized honeybee *A. mellifera*.

## 2. Results

### 2.1. ApAV Protection against Africanized A. mellifera Venom Lethality

**Figure 1:**
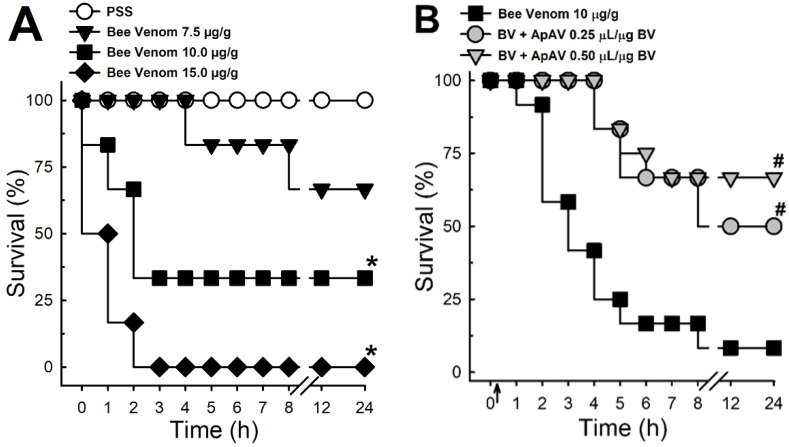
Lethality induced by Africanized *Apis mellifera* venom in mice and treatment with Apilic antivenom. (**A**) Lethality curve produced by different doses of honeybee venom (BV, 7.5, 10 and 15 μg/g, i.p.) in 24 h (n = 6). Log-rank test *p* = 0.0007, * *p* < 0.05 vs. PSS. (**B**) Lethality curve after BV injection (10 μg/g, i.p) and posttreatment (15 min) with Apilic antivenom (ApAV, 0.25 and 0.50 μL/μg BV, i.v.) (n = 6–12). Log-rank test *p* = 0.0017, ^#^ *p* < 0.05 vs. BV.

Africanized *A. mellifera* venom caused the death of mice in a dose-dependent manner (Figure 1A). The dose that induced death at around 50% (10 µg honeybee venom/g mouse) was chosen for the challenge with ApAV. An ApAV i.v. injection 15 min after honeybee venom (10 µg honeybee venom/g mouse) mitigated lethality in both neutralization protocols (0.25 and 0.50 μL ApAV/μg honeybee venom) (Figure 1B). It is worth mentioning that 0.5 μL ApAV/μg honeybee venom reduced honeybee venom lethality by more than 50% after 24 h.

### 2.2. ApAV Inhibition on In Vivo Myotoxicity and Hemoconcentration Induced by Africanized A. mellifera Venom

**Figure 2:**
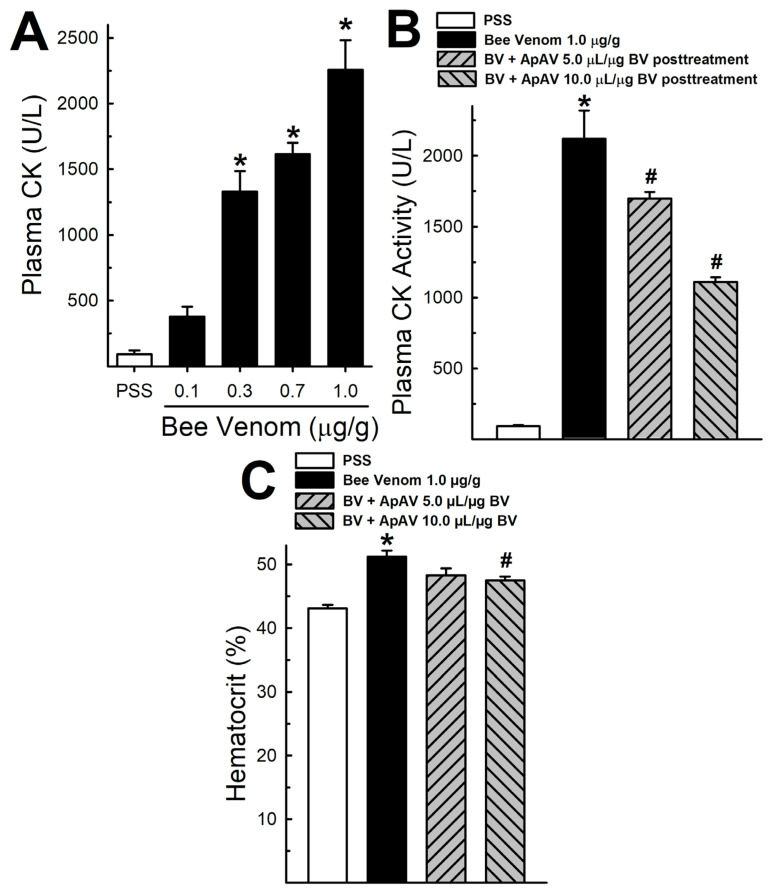
Myotoxic activity and hematocrit alteration induced by Africanized *Apis mellifera* venom and treatment with Apilic antivenom in mice. (**A**) Plasma creatine kinase (CK) activity of mice injected with increasing doses of honeybee venom (BV, 0.1–1.0 μg/g, p.m.) measured after 2 h (n = 3–4). (**B**) Plasma CK activity after p.m. injection of BV (1 μg/g) and posttreatment with Apilic antivenom (ApAV, 5 and 10 μL/μg BV, i.v.) (n = 3–4). (**C**) Hematocrit of mice at 2 h after perimuscular BV injection (1 μg/g) and posttreatment with Apilic antivenom (ApAV, 5 and 10 μL/μg BV, i.v.) (n = 8–10). Data are mean ± SEM. One-Way ANOVA followed by Bonferroni’s post-hoc test (* *p* < 0.05 vs. PSS; ^#^ *p* < 0.05 vs. BV).

The p.m. injection of Africanized *A. mellifera* venom at different doses induced a significant increase in plasma CK activity 2 h after injection of doses higher than 0.3 μg/g (Figure 2A). Intravenous administration of 5 and 10 µL ApAV/µg honeybee venom 15 min after p.m. injection of the venom (1 μg honeybee venom/g mouse) partially blocked this activity by around 25% and 50%, respectively (Figure 2B). A similar profile was observed with the hematocrit of mice evaluated using the same protocol (Figure 2C). In a protocol in which honeybee venom was preincubated with ApAV (1 or 2 µL/µg honeybee venom) for 30 min, ApAV showed higher neutralization of myotoxicity after p.m. injection (see Supplementary Figure S1).

### 2.3. ApAV Inhibition of In Vivo Vascular Permeability Induced by Africanized A. mellifera Venom

**Figure 3:**
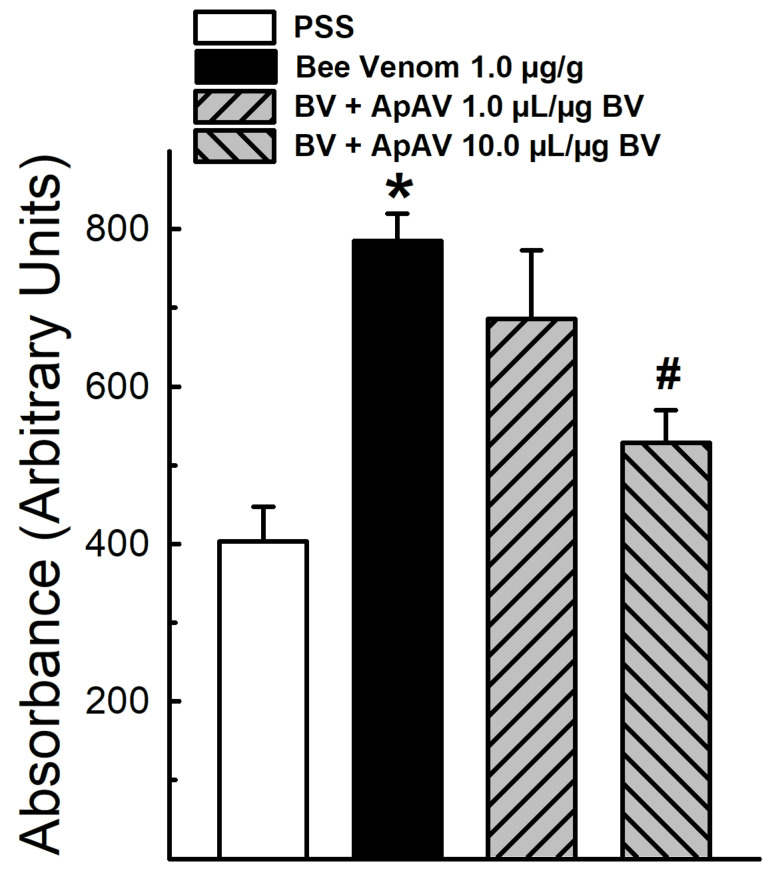
Vascular permeability induced by Africanized *Apis mellifera* venom and treatment with Apilic antivenom in mice. Absorbance of Evans blue dye extravasation in mice after intradermic injection of honeybee venom (BV, 1 μg/g) and posttreatment with Apilic antivenom (ApAV, 1 and 10 μL/μg BV, i.v.) (n = 4). Data are mean ± SEM. One-Way ANOVA followed by Bonferroni’s post-hoc test (* *p* < 0.05 vs. PSS; ^#^ *p* < 0.05 vs. BV).

The intradermic injection of Africanized *A. mellifera* venom increased the capillary leakage of the Evans Blue dye when compared to the group receiving the PSS injection. At least a partial neutralization of honeybee venom effect was observed when animals were treated i.v. with 10 μL ApAV/μg honeybee venom (Figure 3).

### 2.4. ApAV Inhibition of In Vivo Myeloperoxidase Activity Induced by Africanized A. mellifera Venom

**Figure 4:**
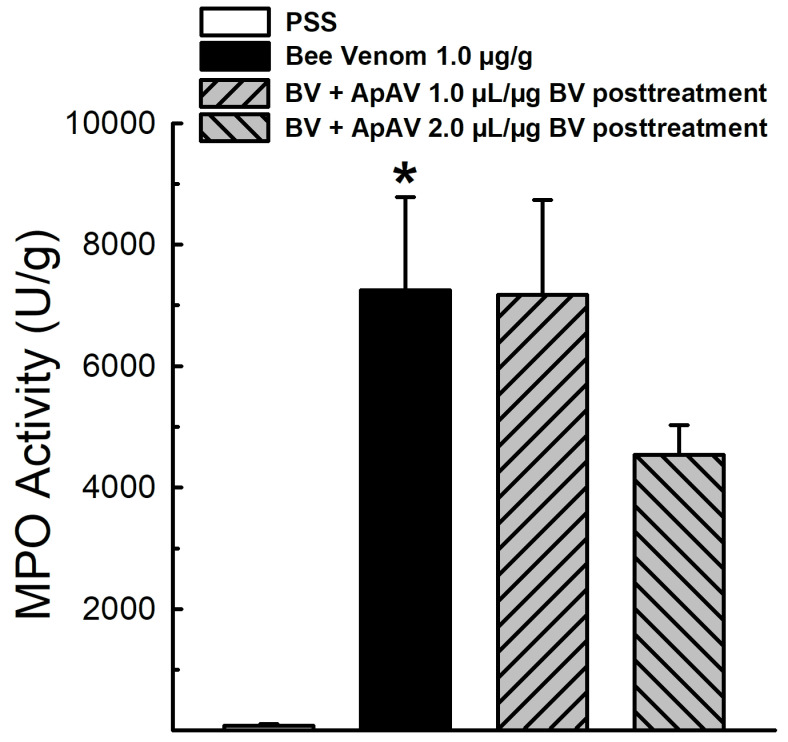
Myeloperoxidase activity induced by Africanized *Apis mellifera* venom and treatment with Apilic antivenom in mice. Myeloperoxidase (MPO) activity of mice receiving injection of honeybee venom (BV, 1.0 μg/g, p.m.) and posttreatment with Apilic antivenom (ApAV, 1.0 and 2.0 μL/μg BV i.v.) (n = 4). Data are mean ± SEM. One-Way ANOVA followed by Bonferroni’s post-hoc test (* *p* < 0.05 vs. PSS).

The p.m. injection of 1 μg/g Africanized *A. mellifera* venom induced a huge augmentation of myeloperoxidase activity in extensor digitorum longus muscle (Figure 4). This elevation may be related to increased neutrophil infiltration and inflammation at the site 6 h after the injection. Intravenous posttreatment with 1 and 2 μL ApAV/μg honeybee venom at 15 min after injection of honeybee venom was not able to reduce myeloperoxidase activity (Figure 4). However, in a second protocol, in which the same doses of honeybee venom and ApAV were preincubated for 30 min and p.m. injected, we observed at least a partial neutralization of honeybee venom-evoked myeloperoxidase activity (Supplementary Figure S2).

### 2.5. ApAV Inhibited Paw Edema Induced by Africanized A. mellifera Venom

**Figure 5:**
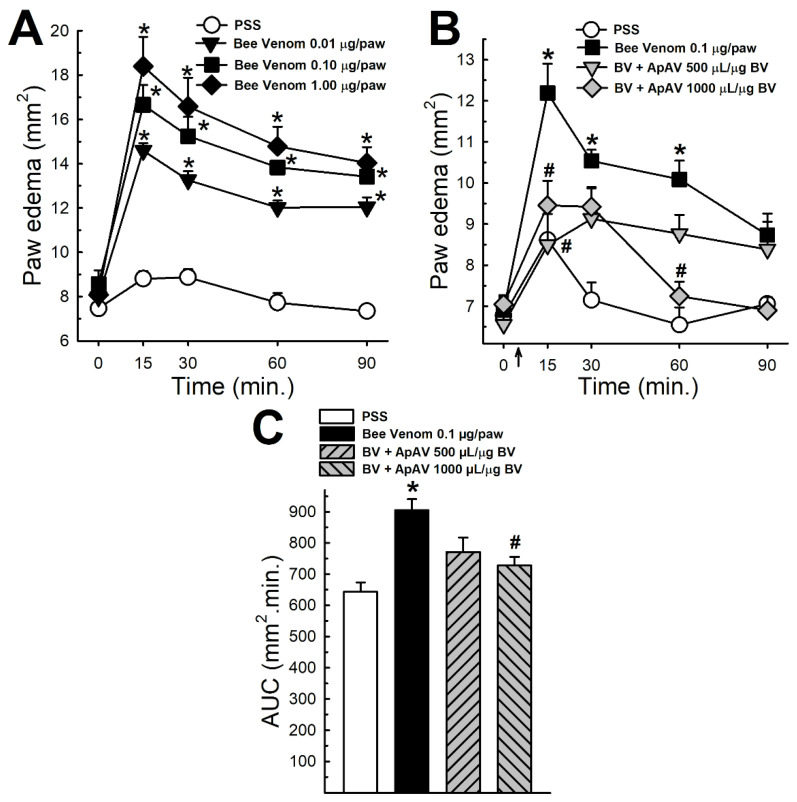
Edematogenic activity of Africanized *Apis mellifera* venom and treatment with Apilic antivenom. (**A**) Time course of paw edema induced by increasing intraplantar doses of honeybee venom (BV, 0.01–1.0 μg/paw) (n = 4). (**B**) Time course of paw edema (0.1 μg BV/paw) and treatment with Apilic antivenom (ApAV, 500 and 1000 μL/μg BV, i.v.) (n = 4) (**C**) Area under the curve from data provided in panel B. Data are mean ± SEM. Two-Way (**A**,**B**) and One-Way (**C**) ANOVA followed by Bonferroni’s post-hoc test (* *p* < 0.05 vs. PSS; ^#^ *p* < 0.05 vs. BV).

Increasing doses (0.01–1 µg honeybee venom/g mouse) of Africanized *A. mellifera* venom injected in the paw of mice induced edema that peaked 15 min after administration at all doses tested (Figure 5A). Edema was much more resistant to ApAV effect than previous in vivo actions. We observed that a statistically significant decline of the edema peak (15 min) occurred in posttreatment with 500 and 1000 µL ApAV/µg honeybee venom (Figure 5B) after honeybee venom paw injection (0.1 µg/paw) and an overall decrease of edema volume followed for 90 min only with 1000 µL ApAV/µg honeybee venom (Figure 5C). ApAV alone (1000 µL ApAV/µg honeybee venom) was not different from PSS (not shown).

### 2.6. ApAV Inhibited Phospholipase and Hyaluronidase Activity of Africanized A. mellifera Venom

**Figure 6:**
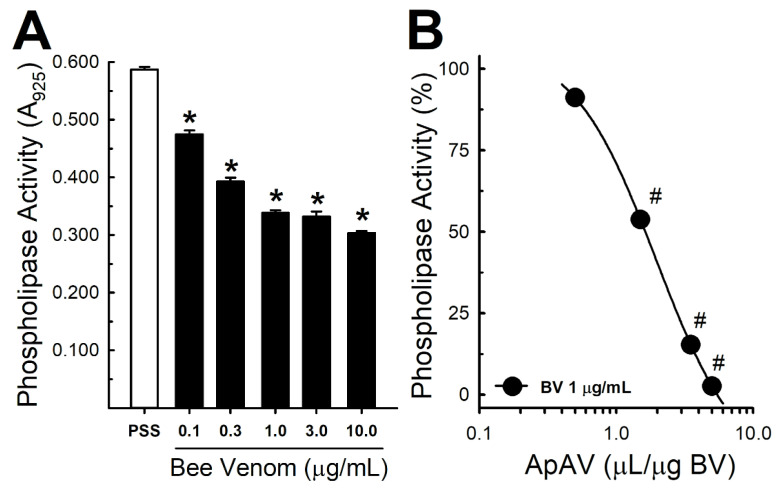
Phospholipase activity of Africanized *Apis mellifera* venom and neutraization by Apilic antivenom. (**A**) Phospholipase activity of increasing concentrations of honeybee venom (BV, 0.1–10.0 μg/mL) (n = 4–16). (**B**) Inhibition curve of honeybee venom phospholipase activity (1 μg/mL) by preincubation with Apilic antivenom (ApAV, 0.5–5.0 µL/µg BV) (n = 6–8). Data are mean ± SEM. One-Way ANOVA followed by Bonferroni’s post-hoc test (* *p* < 0.05 vs. PSS; ^#^ *p* < 0.05 vs. BV).

The addition of Africanized *A. mellifera* venom to a suspension rich in phospholipids decreased the absorbance by reducing its turbidity in a concentration-dependent manner (Figure 6A). ApAV inhibition of honeybee venom phospholipase activity also depended on the concentration with an estimated IC_50_ = 1.63 μL/µg honeybee venom (Figure 6B).

**Figure 7:**
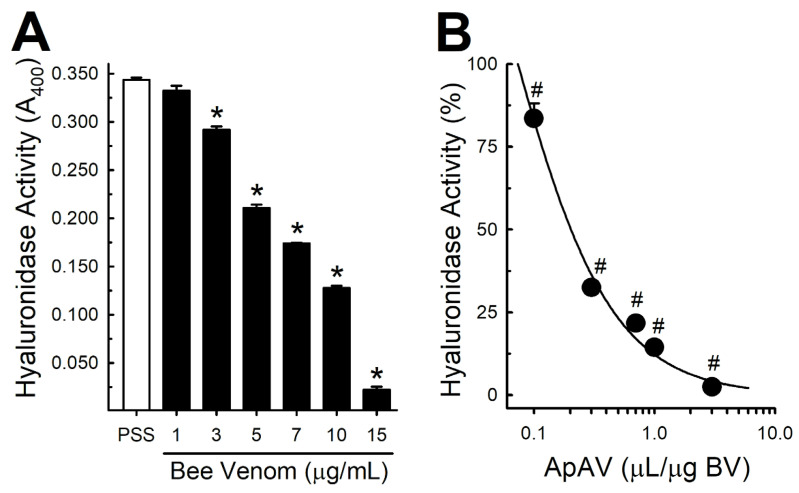
Hyaluronidase activity of Africanized *Apis mellifera* venom and neutralization by Apilic antivenom. (**A**) Hyaluronidase activity of increasing concentrations of honeybee venom (BV, 1–15 μg/mL) (n = 5–10). (**B**) Inhibition curve of honeybee venom hyaluronidase activity (10 μg/mL) by preincubation with Apilic antivenom (ApAV, 0.1–3.0 µL/µg BV) (n = 7–8). Data are mean ± SEM. One-Way ANOVA followed by Bonferroni’s post-hoc test (* *p* < 0.05 vs. PSS; ^#^ *p* < 0.05 vs. BV).

Likewise, Africanized *A. mellifera* venom also decreased in a concentration-dependent manner the absorbance of a solution containing hyaluronic acid as a result of its degradation (Figure 7A). As to the phospholipase activity using a 10-fold higher concentration of honeybee venom (10 µg/mL), ApAV produced a concentration-dependent inhibition of honeybee venom hyaluronidase activity with a lower IC_50_ (0.20 μL/µg honeybee venom) (Figure 7B). 

### 2.7. ApAV Inhibited the Cytotoxic Activity of Africanized A. mellifera Venom and Melittin 

**Figure 8:**
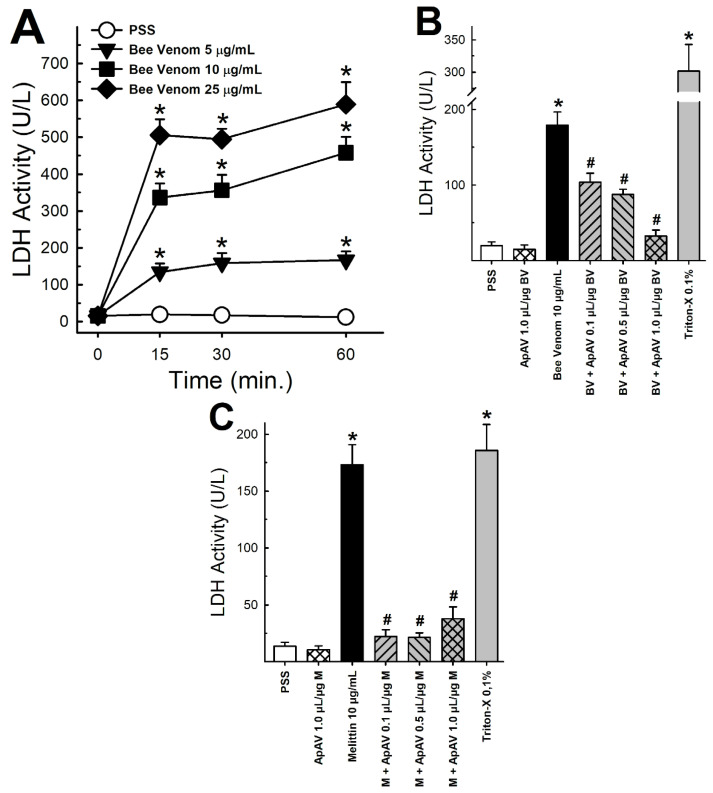
Lactate dehydrogenase (LDH) activity released from LLC-PK1 cells after exposure to Africanized *Apis mellifera* venom or melittin, and neutralization by Apilic antivenom. (**A**) LDH release after exposure of the cells to different concentrations of honeybee venom (BV, 5–25 μg/mL) at different times (15–60 min) (n = 6). (**B**) LDH release after exposure of cells to BV (10 μg/mL) and preincubated with Apilic antivenom (ApAV, 0.1–1.0 μL/μg BV) for 15 min (n = 5–12). (**C**) LDH release after exposure to melittin (10 μg/mL) and preincubated with Apilic antivenom (0.1–1.0 μL/μg M) for 15 min (n = 6). Data are mean ± SEM. One-Way (B, C) and Two-Way (A) ANOVA followed by Bonferroni’s post-hoc test (* *p* < 0.05 vs. PSS; ^#^ *p* < 0.05 vs. BV or M).

Africanized *A. mellifera* venom promoted a concentration- and time-dependent increase of LDH release in renal LLC-PK1 culture medium, indicating cell injury (Figure 8A). Subsequently, when we preincubated with 0.1–1.0 μL ApAV/µg honeybee venom for 15 min, we observed a reduction of this activity (Figure 8B). Since melittin is the major component of Africanized *A. mellifera* venom, we also evaluated its activity. Melittin induced an increase of LDH release in LLC-PK1 cells, comparable to the whole venom, and its effect was blocked by ApAV as well (Figure 8C).

**Figure 9:**
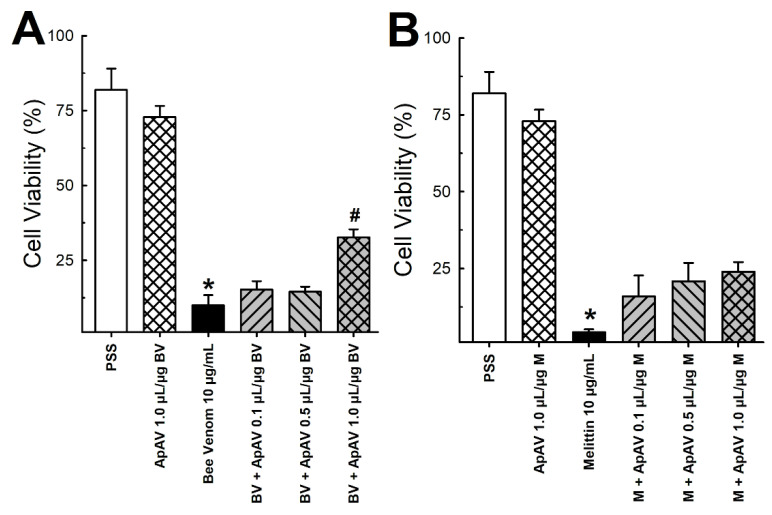
LLC-PK1 cell viability after exposure of Africanized *Apis mellifera* venom or melittin and neutralization by Apilic antivenom. (**A**) Cell viability in the presence of honeybee venom (BV, 10 μg/mL) and pre-incubated with Apilic antivenom (ApAV, 0.1–1.0 µL/µg BV) (n = 3). (**B**) Cell viability in the presence of melittin (M, 10 μg/mL) and pre-incubated with Apilic antivenom (ApAV, 0.1–1.0 µL/µg BV) (n = 3). Data are mean ± SEM. One-Way ANOVA followed by Bonferroni’s post-hoc test (* *p* < 0.05 vs. PSS; ^#^ *p* < 0.05 vs. BV or M).

**Figure 10:**
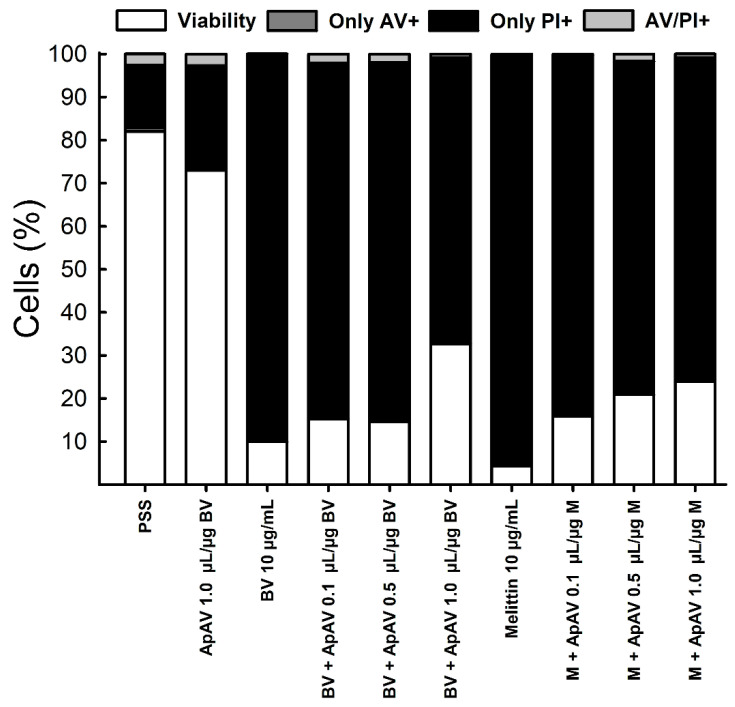
Profile of viable, necrotic, apoptotic and late apoptotic LLC-PK1 cells after exposure to Africanized *Apis mellifera* venom or melittin and neutralization by Apilic antivenom. Cells exposed to 10 μg/mL whole honeybee venom (BV) or melittin (M) alone or in the presence of increasing concentrations of Apilic antivenom (ApAV, 0.1–1.0 µL/µg BV or M) were evaluated by flow cytometry. The average percentages of viable (white), apoptotic (annexin V positive–AV+, dark grey), necrotic (propidium iodide positive–PI+, black) and late apoptotic (AV/PI+, light grey) cells are shown (n = 3).

Using the same protocol, the effect of Africanized *A. mellifera* venom and melittin on LLC-PK1 cells was analyzed by flow cytometry, which revealed a large loss of cell viability after exposure for 15 min (Figure 9A,B). Cells essentially died by necrosis (Figure 10). Although less remarkable than in the LDH assay, protection from cytotoxicity was significantly achieved by ApAV (Figure 9A,B and Figure 10). 

### 2.8. ApAV Inhibited the Hemolytic Activity of the Africanized A. mellifera Venom and Melittin

**Figure 11:**
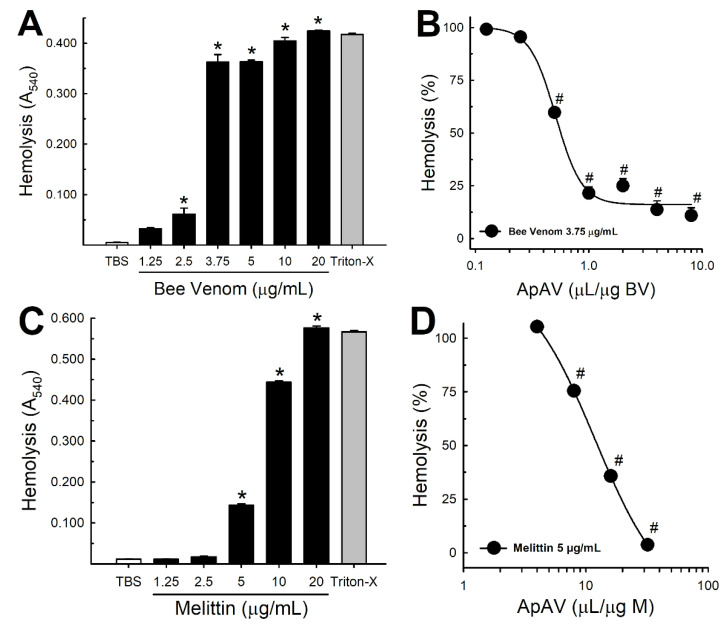
Hemolytic activity of Africanized *A. mellifera* venom and melittin and inhibition by Apilic antivenom. (**A**) Hemolysis curve produced by increasing concentrations of honeybee venom (BV, 1.25–20 μg/mL) (n = 5). (**B**) Percentage of hemolysis produced by honeybee venom (3.75 μg/mL) in the presence of increasing volumes of Apilic antivenom (ApAV, 0.125–8 μL/μg BV) (n = 5–25). (**C**) Hemolysis curve produced by increasing concentrations of melittin (1.25–20 μg/mL) (n = 5). (**D**) Percentage of hemolysis produced by melittin (5 μg/mL) in the presence of increasing volumes of Apilic antivenom (ApAV, 4–32 μL/μg M) (n = 5–10). Data are mean ± SEM. One-Way ANOVA followed by Bonferroni’s post-hoc test (* *p* < 0.05 vs. PSS; ^#^ *p* < 0.05 vs. BV or M).

Consistent with the necrotic, plasma membrane lytic effect on LLC-PK1 cells, Africanized *A. mellifera* venom and melittin promoted significant hemolysis of murine erythrocytes starting from 2.5 and 5 µg/mL, respectively (Figure 11A,C). The hemolytic effect was gradually inhibited after preincubation with ApAV (Figure 11B,D), with lower IC_50_ to neutralize honeybee venom (0.57 µL/µg honeybee venom) than melittin (12.5 µL/µg melittin).

## 3. Discussion

Here, we demonstrated that *Apis mellifera* venom causes several in vivo manifestations, such as edema, myotoxicity, hemoconcentration, increased capillary permeability and, eventually, death in mice. In addition to these actions, we observed increased myeloperoxydase, phospholipase A2 and hyaluronidase activity in vitro. Direct cytotoxicity by crude venom and melittin toxin in renal proximal tubular cells (LLC-PK1) was also seen. Apilic antivenom inhibited and/or neutralized practically all of these actions caused by *A. mellifera* venom and that of the toxin melittin, and come up as an effective therapeutic agent for the use in events caused by bee stings.

This new Apilic antivenom is produced only against the main Africanized honeybee venom toxins that do not cause pain to animals [27]. Its use in patients affected by multiple stings was only possible in association with a robust clinical protocol [14]. The results demonstrated that several in vitro and in vivo activities of the Africanized *A. mellifera* venom, including melittin, its main constituent, could be partially or almost fully neutralized by ApAV. This antivenom was developed only with the main toxins from the Africanized honeybee venom. Thus, the treatment of patients with ApAV includes a robust clinical protocol along with several drugs [14].

The mortality rate induced by the honeybee venom, evaluated in mice, was similar to what we found previously [28]. ApAV delayed the time animals died and protected them from death after 24 h (eight-fold increase in survival rate with 0.5 μL ApAV/μg honeybee venom). This result is important since the main pharmacological tool for assessing the quality of a polyclonal antivenom is its ability to inhibit venom-induced lethality [29]. For instance, Pessenda et al. [30] demonstrated that human monoclonal antibody fragments produced against melittin and PLA_2_ (Afribumab) exhibited incomplete protection in lethality protocols; indeed, lethality was not reduced, only delayed. Because Afribumab was preincubated with honeybee venom for biological evaluation, we consider our results closer to the clinical situation.

The most common clinical manifestation after honeybee attack is the inflammatory reaction, with a massive release of pro-inflammatory cytokines (IL-1, IL-6, TNF-α) that increase vascular permeability with the consequent loss of volume for the third space [6,31]. The effect of ApAV on the inflammatory process was investigated by the mouse paw edema test. Honeybee venom induced significant edema at a dose as low as 0.01 μg/g. ApAV reduced the peak as well as the total edema response after intravenous injection of honeybee venom at 1000 μL/μg. In this case, the ApAV dose needed was considerably higher than those required for other in vivo activities (500 and 1000 μL ApAV/μg honeybee venom), possibly due to the difficulty of distributing ApAV to poorly perfused edematous tissue [32]. 

Edema is part of the inflammatory reaction and it is derived from aberrant capillary permeability, which we examined after an intradermal injection of honeybee venom in mouse abdomen skin. At 10 μL ApAV/μg honeybee venom, intravenous ApAV partially prevented the increase of vascular permeability. Hemoconcentration is also an event related to increased blood vessel permeability. Accordingly, we have shown the hematocrit increase after honeybee venom administration [28]. As in the case of the capillary permeability test, hemoconcentration promoted by honeybee venom injection was partially inhibited by 10 μL ApAV/μg honeybee venom. This result corroborates an important mechanism to reduce lethality induced by bee venom, since this systemic effect is associated with hypovolemic shock as a mechanism of death by honeybee attack [28]. 

Perimuscular injection of the venom was employed to evidence another inflammatory marker, myeloperoxidase [33]. Neutrophil myeloperoxidase activity showed a remarkable elevation, similar to *Bothrops* venom as demonstrated earlier [34,35]. ApAV was unable to neutralize this inflammatory effect of honeybee venom when administered afterwards, but it partially affected the activity when incubated before injection. Myotoxicity was assessed indirectly by measuring increase of plasma CK activity after p.m. injection, together with the inflammatory reaction and neutrophil migration [35]. It has been demonstrated that intravenous administration of honeybee venom promotes the increase of plasma CK activity in rats [36]. ApAV also inhibited the increase of plasma CK activity when preincubated with whole honeybee venom. Funayama et al. [37] reported that human monoclonal single-chain Fv (scFV) was also able to inhibit the increase of plasma CK activity when preincubated with whole honeybee venom. As we previously described, the increase of plasma CK in mice reach a peak at 2 h after intramuscular injection and reflects the sarcolemma damage of skeletal muscle fibers, that we ascribe as myotoxicity [28]. We also showed that isolated mouse EDL or soleus muscle exposed to honeybee venom presents a quick and intense injury, revealed by the rate of CK release [38]. 

In the clinical setting, rhabdomyolysis is regarded as the major cause of kidney damage induced by honeybee venom [23]. Although this venom is injected into the skin, the systemic effect can lead to muscle damage and the released myoglobin would cause renal failure [23]. Myoglobin affects the brush border of proximal contorted tubules and stimulates tubulointerstitial damage, leading to acute tubular necrosis [23]. Dos Reis et al. [39] demonstrated the occurrence of morphological alterations corresponding to cellular lysis in the proximal tubule of animals after intravenous injection of honeybee venom, indicating its interaction with the plasma membrane and rhabdomyolysis, which could precipitate renal failure. Thus, the reduction of myotoxicity in the protocol of p.m. venom injection is beneficial as a mechanism of protection against muscle damage and, possibly, of kidney damage induced by this venom. On the other hand, Han et al. [40] demonstrated the ability of melittin to reduce cell viability and increase LDH release from rabbit proximal contorted tubule cells, as well as interfere with cellular transport ability by reducing the uptake of sodium, inorganic phosphate and α-methyl-D-glucopyranoside. In the present study, we demonstrated that honeybee venom and melittin diminished pig renal proximal tubule epithelial cell viability by necrosis while ApAV hampered cell death. Thus, ApAV is a putative nephroprotective treatment both by reducing myotoxicity and directly avoiding renal cell damage.

Comparable to the lytic effect and protection observed in renal cells, the hemolysis protocol evidenced that both whole venom and melittin caused red cell lysis. An interesting observation is that the IC_50_ for hemolysis was lower with the whole venom when compared to melittin. Therefore, the amount of melittin present in the whole venom at the concentration used (about 0.4 μg) would be unable to cause hemolysis alone. This fact reveals the importance of combining the different components of honeybee venom with melittin, especially PLA_2_ [41]. Consistently, ApAV inhibited honeybee venom phospholipase and hyaluronidase activities, which contribute to the cellular damage, and stimulate the inflammatory process and the systemic spread of the venom after injection [42]. In addition, it is reported that the hyaluronidase present in honeybee venom is the major component responsible for the allergenic response induced by this venom, whereas in our results the ApAV decreased the inflammatory response [43]. Recently, a group of investigators produced scFv antibody fragments that protected mice from the venom myotoxicity as well as against the lethal dose of honeybee venom. These protective effects were potentiated when they combined the antibodies. They demonstrated that these monoclonal antibodies (Afribumab), produced specifically against melittin and PLA_2_, could antagonize the separate activities of melittin, PLA_2_ and the whole honeybee venom. They described that Afribumab 1 and 2 alone reduced the cytotoxic activities of honeybee venom components, and the mixture of both antibodies improved this inhibition [30,37]. Most of these observations are in agreement with our present data, showing the necessity of developing a specific treatment, a safe agent that can counteract and stop the deleterious effects caused by a massive, Africanized honeybee attack, which is a health problem in Brazil and other countries in the Americas [6,8,13,14,15,16,17].

In conclusion, it was demonstrated that the ApAV is pharmacologically effective in reducing the various in vivo and in vitro effects of Africanized honeybee venom (*A. mellifera*) and melittin. Thus, this Apilic antivenom emerges as a promising immunobiological product for the treatment of massive, Africanized honeybee attacks.

## 4. Materials and Methods 

### 4.1. Animals and Reagents

In this study, we used 130 adult Swiss mice of both sexes to study the lethality, hemorrhagic effect, edema myotoxicity and inflammatory response in vivo. These animals were provided by the Rodent Vivarium at the Institute of Microbiology Paulo de Goes and the Federal University of Rio de Janeiro. The mice (20–25 g) used for the study were housed in a 12/12 h light/dark cycle at room temperature of 25 °C, humidity level of 60–70% with access to food and water *ad libitum*. One coauthor (MAS), a veterinary doctor, monitored the animal health to certify that there were no grimaces or other signs of pain, changes in the level of activity, locomotion or abnormal behavior throughout the experiments in all groups. However, unlike other poisons used in our laboratory, mice administered Africanized *A. mellifera* venom did not exhibit clear clinical endpoints to proceed euthanasia during the experiment. Animals were euthanized with isoflurane overdose immediately after each protocol at predeterminated times. Overall, 30 animals died without being euthanized.

All animal experimentation protocols were approved by the Ethics Committee for Animal Experimentation (Health Sciences Center, Federal University of Rio de Janeiro–UFRJ) (protocol no. DFBCICB072-04/16), in agreement with Brazilian federal law (11.794/2008, Decree Number 6.899/2009) on 27 June 2017. We followed institutional guidelines on animal manipulation, adhering to the ‘‘Principles of Laboratory Animal Care’’ (National Society for Medical Research, Chicago, IL, USA) and the ‘‘Guide for the Care and Use of Laboratory Animals’’ (National Academy of Sciences, Washington, DC, USA).

Africanized *A. mellifera* venom was obtained from the Comissão Executiva do Plano da Lavoura Cacaueira (Executive Committee of the Cacao Plantation Plan, CEPLAC, Bahia state, Brazil). Melittin toxin, Dulbecco’s Modified Eagle Medium (DMEM), Triton X-100, hexadecyltrimethylammonium bromide (HTAB) and *o*-dianisidine were purchased from Sigma Chemical Co, St. Louis, MO, USA. Fetal bovine serum (FBS) and trypsin-EDTA solution were obtained from Invitrogen, Waltham, MA, USA. Creatine kinase activity (CK) and lactate dehydrogenase activity (LDH) were determined, respectively, using a CK NAC kit and LDH UV kit, both from Bioclin, whereas hyaluronic acid was purchased from Fluka, USA. Physiological saline solution (PSS: NaCl 135 mM; KCl 5 mM; CaCl_2_ 2 mM; MgCl_2_ 1 mM; NaH_2_PO_4_ 1 mM), phosphate buffered saline (PBS) and Tris-buffered saline (Tris-HCl 10 mM pH 7.5 + NaCl 150 mM) were also used in some of our experiments.

ApAV-developed by the researchers of the Center for the Study of Venoms and Venomous Animals (CEVAP) of the São Paulo State University (UNESP) in partnership with the Vital Brazil Institute (IVB), Brazil-was donated by CEVAP and IVB to conduct these experiments. ApAV is composed of F(ab’)2 fraction of specific immunoglobulins contained in heterologous and hyperimmune serum from the blood of horses previously immunized only with the venom toxins. ApAV development and clinical trial protocols are detailed in the patent [27] and in Barbosa et al. [14]. The batch of antivenom used in our experiments was 155803 A. According to Brazilian and WHO Guidelines, serum antivenom potency is assessed by a lethality protocol including preincubation of ApAV with honeybee venom. It was specified that each 1 mL of ApAV neutralizes 1.25 mg of whole honeybee venom. Thus, different variations of this antivenom ratio were utilized to neutralize honeybee venom/melittin in our protocols. All quality control tests were within WHO specifications, while the whole protein content of this ApAV batch (155803 A) was 3.6%, which does not exceed the maximum allowed by the WHO (10%) [29].

### 4.2. Cell Line

Immortalized porcine (*Sus scrofa*) renal proximal tubule epithelial cell line LLC-PK1 (ATCC CL-101™, American Type Culture Collection) was grown on culture plates in DMEM supplemented with 10% FBS, NaHCO_3_ (44 mM) and gentamicin (40 mg/L) (pH 7.4) at 37 °C and 5% CO_2_. When confluent, cells were passed using 0.25% trypsin-EDTA solution. Experiments were performed with cells at a confluency of approximately 80%, and the medium was changed to DMEM without FBS on the day before the experiment [44].

### 4.3. Lethality

The systemic effect of Africanized *A. mellifera* venom was evaluated by the lethality test in mice. Different groups of animals (n = 6) received intraperitoneal (i.p.) injection of increasing doses of honeybee venom (7.5–15 μg/g) to evaluate the lethality profile. In a subsequent experiment, the animals received 10 μg/g venom i.p. and after 15 min they were treated with intravenous (i.v.) injection of 0.25 and 0.5 μL ApAV/μg honeybee venom (n = 6–12). All animals were observed for a 24 h period and the survivors were quantified [28].

### 4.4. Myotoxicity In Vivo and Hematocrit

In vivo myotoxicity was assessed after a perimuscular (p.m.) injection of Africanized *A. mellifera* venom (0.1 to 1 μg/g) into the right hind limb of the animal as previously described [45,46,47,48]. The venom was diluted in PSS and injected at the volume of 50 μL per animal. The treatment was performed by i.v. injection (250 μL) of 5 or 10 μL ApAV/μg honeybee venom 15 min after honeybee venom injection. After 2 h, the animals were anesthetized with isoflurane and blood collected by orbital puncture in heparinized microcapillaries. The plasma was separated by centrifugation and the hematocrit was analyzed using a percentage hematocrit ruler. The determination of plasma creatine kinase (CK) activity was performed as previously described [28,49,50]. In addition, CK activity was expressed in international units (U), where 1 U is the amount that catalyzes the transformation of 1 μmol of substrate at 25 °C.

### 4.5. Vascular Permeability

Plasma extravasation from mouse skin vessels induced by Africanized *A. mellifera* venom was evaluated through i.v. injection of Evans Blue dye (100 μL of a 2.5% solution), according to previous studies [28,51]. After 15 min, the animals received intradermal (i.d.) injection of honeybee venom (1 μg/g) into the abdomen in a final volume of 100 μL. The treatment protocol was performed immediately after by the i.v. administration of 1 and 10 μL ApAV/μg honeybee venom. After 30 min, the animals were sacrificed under anesthesia. Then, the skin of the abdomen was removed and maintained at room temperature for 72 h. The skin was then fixed to a Lucite base plate, and the entire area at the injection site and surrounding area was transilluminated using incandescent light. Light transmitted over a skin area of 109 mm^2^ was read and light transmission or absorbance was normalized by taking the mean readings of each group with the values being expressed as arbitrary units of absorbance, as previously described [28,51].

### 4.6. Myeloperoxidase Activity

To determine myeloperoxidase activity (MPO), animals received 100 μL p.m. injection of 1 μg/g of Africanized *A. mellifera* venom. After 15 min, the animals were treated intravenously with 1 or 2 μL ApAV/μg honeybee venom. A second protocol was performed, where the animals received p.m. injection of 1 μg/g honeybee venom pre-incubated with 1 or 2 μL ApAV/μg honeybee venom for 30 min at 37 °C. After 6 h, mice were euthanized under anesthesia, and muscles were removed, freed from fat and tendons, dried with absorbent paper and weighed. To evaluate muscle MPO content, muscles were homogenized in 1 mL hexadecyltrimethylammonium bromide (HTAB) (5 mg/mL) solution, from which 100 μL were added to 900 μL of o-dianisidine 1 μM + 0.001% H_2_O_2_ solution to measure MPO activity for 3 min with 30 s intervals at 460 nm. Results were expressed as enzyme units per gram of muscle tissue (U/g). The injection of PSS alone was used as the negative control [52].

### 4.7. Paw Edema

Edema induction was evaluated by 10 μL intraplantar injection of 0.01 to 1 μg Africanized *A. mellifera* venom/paw. Treatment was evaluated by i.v. injection of 500 or 1000 μL ApAV/μg honeybee venom at 5 min after 0.1 μg honeybee venom/paw intraplantar injection. The height and width of the paw were measured using a caliper rule and the product of the values was the area, with the edema being expressed in mm^2^. The area under the curve of paw edema versus time, for 90 min after venom injection and treatments, was determined by the trapezoidal rule and employed to quantify the data [28].

### 4.8. Honeybee Venom Phospholipase Activity

The phospholipase activity was determined using the modified turbidimetric method described by Marinetti [28,53]. First, a stock solution was prepared using a fresh egg yolk, which was separated, filtered and diluted in 150 mM NaCl until achieving a volume of 100 mL. From the stock solution, a work solution was prepared after 1:10 dilution in saline. Each well was filled with a solution contained 4 µL CaCl_2_ 0.5 M (final concentration of 10 mM), 5 µL sodium taurocholate 0.4% (final concentration of 0.01%), 5 µL Tris-HCl 0.2 M (pH 7.5, final concentration of 5 mM), 60 µL of work solution and sufficient saline to complete a final volume of 200 µL. The volume corresponding to Africanized *A. mellifera* venom (1 µg/mL) alone or preincubated for 30 min at 37 °C with ApAV (0.5–5.0 µL/µg honeybee venom) was subtracted from saline. The reaction occurred at 37 °C for 30 min and absorbance was measured by ELISA at 925 nm immediately and 30 min after addition of substances. The results were expressed as percentage of change in absorbance compared with the venom group.

### 4.9. Honeybee Venom Hyaluronidase Activity

Hyaluronidase activity was assessed by using the Di Ferrante method [54]. Bovine hyaluronic acid (Fluka, Charlotte, NC, USA) was used as a substrate to evaluate the reduction of the solution’s turbidity. Hyaluronic acid was reconstituted with acetate buffer (0.2 M acetic acid + 0.15 M NaCl pH 6.0) solution and added into an ELISA 96-well microplate (final concentration of 100 μg/mL). Africanized *A. mellifera* venom (1–15 μg/mL) or the combination of Africanized *A. mellifera* venom (10 μg/mL) plus ApAV (0.1–3.0 μL/µg honeybee venom) was added after 30 min of incubation at 37 °C and completed with buffer up to 100 μL final volume. The solution was kept in a water bath at 37 °C for 60 min. Finally, 200 μL of 2.5% alkyltrimethylammonium bromide (cetrimide) solution was added, and after 10 min at room temperature, the absorbance was measured in an ELISA spectrophotometer at 400 nm. The acetic acid-acetate buffer was used as a blank tube, and the results were expressed as percentage of venom activity.

### 4.10. LDH Assay

For in vitro determination of cytotoxicity, LDH release into the extracellular medium was measured using an LDH activity kit. The assay was performed according to the manufacturer’s instructions. LLC-PK1 cells were cultured in 24-well plates and the medium was changed to phosphate buffered saline (PBS) immediately before the experiment. For time-course assay, cells were exposed to Africanized *A. mellifera* venom (5, 10 and 25 µg/mL) for 60 min. To determine the protective effect of ApAV, 10 μg/mL Africanized *A. mellifera* venom or melittin was preincubated for 30 min at 37 °C with 0.1-1 μL ApAV/μg honeybee venom or melittin, added to cell medium and evaluated after 15 min. LDH activity in supernatants that were recovered and centrifuged was determined by spectrophotometer at 340 nm. Triton X-100 (0.1% solution) was used as a positive control [44]. 

### 4.11. Cell Death Analysis

LLC-PK1 cells were grown in 12-well plates and medium was changed by PBS immediately before the experiment. Cells were exposed to Africanized *A. mellifera* venom (10 μg/mL) or melittin (10 μg/mL) in the presence or absence of 0.1–1 μL ApAV/μg honeybee venom or melittin for 15 min. Apoptosis was evaluated through the analysis of phosphatidylserine exposure: Cells were incubated with annexin V binding buffer containing 10 mM HEPES (4-(2-hydroxyethyl)-1-piperazineethanesulfonic acid), 140 mM NaCl, 2.5 mM CaCl_2_ and 0.75 mM MgCl_2_ at pH 7.4 and labeled with annexin V conjugated to fluorescein-dextran isothiocyanate (FITC) (1:50) for 20 min at room temperature in the absence of light. After incubation, propidium iodide (PI, 10 μg/mL in binding buffer, Sigma-Aldrich, St. Louis, MO, USA) was added to assess necrosis, and then the cells were analyzed by flow cytometry (Accuri C6^®^, BD Bioscience, USA) for the identification of cells with annexin V- and/or PI-positive events [55].

### 4.12. Hemolytic Activity

*A. mellifera* venom or melittin was evaluated by the adapted method described by Fischer et al. [56]. Blood from mice was collected with heparinized microcapillaries under isoflurane anesthesia. After collection, it was centrifuged at 3200 rpm at 20 °C for 3 min and washed 3 times with ice-cold Tris buffer solution (TBS, Tris HCl 10 mM, pH 7.5, NaCl 150 mM), and centrifuged again. Red blood cells were collected and a 2% suspension in TBS was prepared. The protocol consists of 100 μL of this red cell suspension added to 100 μL of TBS alone (negative control) or in the presence of Africanized *A. mellifera* venom (1.25–20 μg/mL) or melittin (1.25–20 μg/mL) alone or in association with ApAV (0.125–32 μL/µg honeybee venom). The reaction occurred for 15 min in a water bath at 37 °C, and then centrifuged at 3200 rpm for 3 min. Supernatant was collected (150 μL) and added to 250 μL of TBS and read at 540 nm in a spectrophotometer. The positive control used was 100 μL of 1% Triton X-100 added to the red cell suspension. The results were described considering honeybee venom or melittin as 100% hemolytic activity.

### 4.13. Statistical Analysis

Data were expressed as mean ± standard error of the mean (SEM). A one-way or two-way analysis of variance (ANOVA) was used to compare groups with one variable or two variables, respectively, followed by Bonferroni’s post-hoc test. The mean of each group was compared with the mean of every other group. Log-rank test was employed to analyze survival curves. The *p* value < 0.05 was used to indicate a significant difference between means.

## Data Availability

The raw data generated and analyzed during the current study are available from the corresponding author I.D. on reasonable request.

## Supplementary Materials

The following are available online at https://www.mdpi.com/2072-6651/13/1/30/s1, Figure S1: Myotoxic activity alteration induced by Africanized *A. mellifera* venom and pretreatment with Apilic antivenom in mice, Figure S2. Vascular permeability induced by Africanized *Apis mellifera* venom and pretreatment with Apilic antivenom in mice.
