# Supplementary material for: A Novel Apilic Antivenom to Treat Massive, Africanized Honeybee Attacks: A Preclinical Study from the Lethality to Some Biochemical and Pharmacological Activities Neutralization
Source: Toxins (Basel). 2021 Jan 5;13(1):30. doi: 10.3390/toxins13010030 (PMC7824798; doi:10.3390/toxins13010030)
Supplement: Supplementary file 1 [file toxins-13-00030-s001.pdf]

Supplementary Materials: A Novel Apilic Antivenom to Treat Massive, Africanized Honeybee Attacks: a Preclinical Study from the Lethality to Some Biochemical and Pharmacological Activities Neutralization

Jhonatha da Mota Teixeira-Cruz, Marcelo Abrahão Strauch, Marcos Monteiro-Machado, Matheus da Silva Tavares-Henriques, João Alfredo de Moraes, Luís Eduardo Ribeiro da Cunha, Rui Seabra Ferreira Jr., Benedito Barraviera, Luis Eduardo M. Quintas and Paulo A. Melo

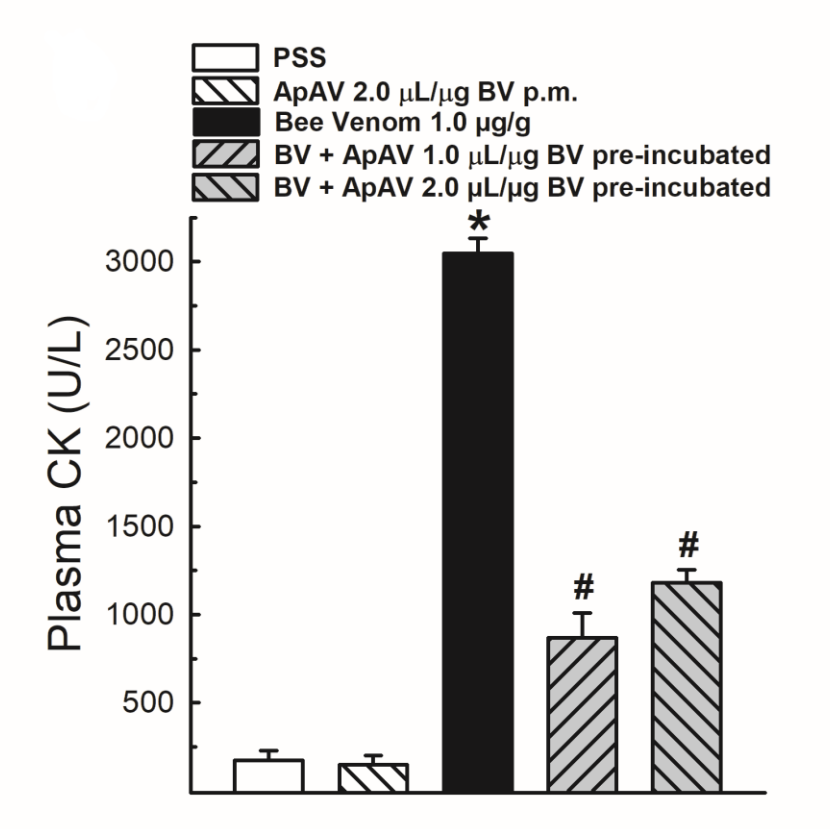

Figure S1. Myotoxic activity alteration induced by Africanized *A. mellifera* venom and pretreatment with apilic antivenom in mice. Plasma creatine kinase (CK) activity after perimuscular injection of honeybee venom (BV, 1 μg/g) pre-incubated with apilic antivenom (ApAV, 1 and 2 μL/μg BV, p.m.) (n = 4). Data are mean ± SEM. One-Way ANOVA followed by Bonferroni's post-hoc test (*$p < 0.05$ *vs.* PSS; #$p < 0.05$ *vs.* BV).

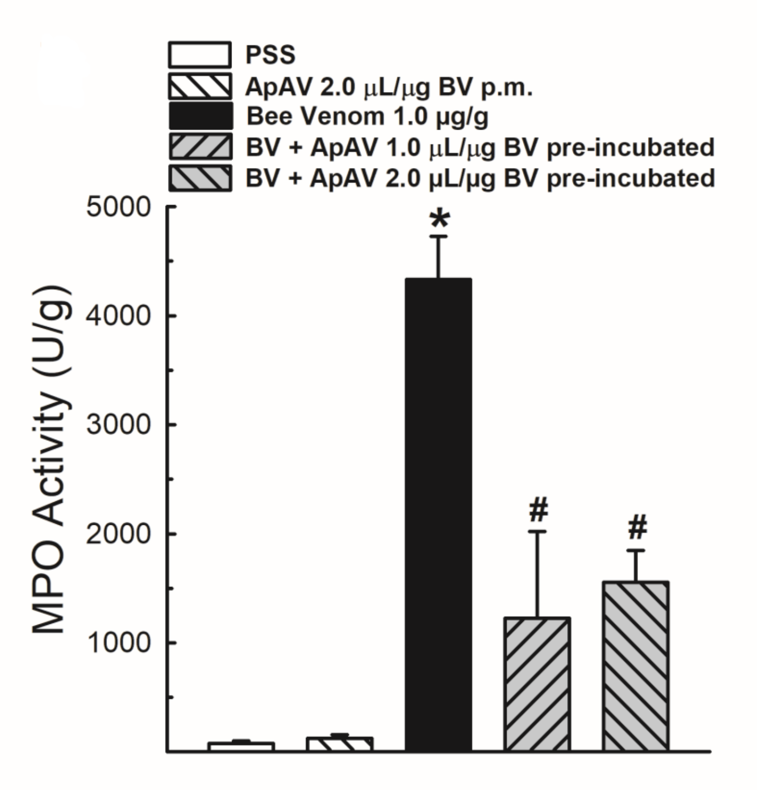

Figure S2. Vascular permeability induced by Africanized *Apis mellifera* venom and pretreatment with apilic antivenom in mice. Absorbance of Evans blue dye extravasation in mice after perimuscular injection of honeybee venom (BV, 1 μg/g) preincubated with apilic antivenom (ApAV, 1 and 10 μL/μg BV, i.v.) (n = 4). Data are mean ± SEM. One-Way ANOVA followed by Bonferroni's post-hoc test (*$p < 0.05$ *vs.* PSS; $^{\#}p < 0.05$ *vs.* BV).
